# Editorial: Metabolomics and transcriptomics in biomarker discovery: mass spectrometric techniques in volatilome research

**DOI:** 10.3389/fmolb.2024.1545016

**Published:** 2025-01-17

**Authors:** Andras Szeitz, Jens Herbig, Konstantinos A. Kouremenos, Shane Fitzgerald, Steven J. Hallam

**Affiliations:** ^1^ Genome Science and Technology Program, University of British Columbia, Vancouver, BC, Canada; ^2^ Department of Microbiology and Immunology, University of British Columbia, Vancouver, BC, Canada; ^3^ Life Sciences Institute, University of British Columbia, Vancouver, BC, Canada; ^4^ IONICON Analytik Gesellschaft m.b.H., Innsbruck, Austria; ^5^ NutriPATH Integrative and Functional Pathology Services, Melbourne, VIC, Australia; ^6^ Insight Science Foundation Ireland Research Centre for Data Analytics, National Centre for Sensor Research, School of Chemical Sciences, Dublin City University, Dublin 9, Ireland; ^7^ Graduate Program in Bioinformatics, University of British Columbia, Vancouver, BC, Canada; ^8^ Department of Biochemistry, Chulalongkorn University, Bangkok, Thailand; ^9^ Bradshaw Research Institute for Minerals and Mining (BRIMM), University of British Columbia, Vancouver, BC, Canada; ^10^ ECOSCOPE Training Program, University of British Columbia, Vancouver, BC, Canada

**Keywords:** volatile organic compounds (VOCs), biomarkers, omics, mass spectrometry, historical review

The central dogma of molecular biology traces a path from genomic sequence information represented in DNA to transcribed messenger RNA decoded at the ribosomal level into proteins that perform a variety of catalytic and regulatory roles within cells (Crick, 1970). The combined activity of these proteins gives rise to pools and fluxes of metabolites within emergent metabolic networks (Durek and Walther, 2008). This genotype to phenotype continuum remains both a fundamental organizing principle for the life sciences and a compelling challenge for systems level thinking in the 21st century (Benfey and Mitchell-Olds, 2008; Costanzo et al., 2019). While our capacity to predict and annotate protein coding potential within genomes and transcriptomes has grown exponentially since the initial introduction of the chain termination method by Sanger and colleagues more than five decade ago (Sanger et al., 1977), it has proven more difficult to constrain the diversity of metabolites over time and space or in relation to health and disease states (Sharon et al., 2014; Sieber and Spradling, 2017).

Nevertheless, researchers have pressed on to profile metabolite pools and fluxes not only within organismal systems, but also within natural and engineered environments, integrating multi-omic (DNA, RNA, protein, and metabolite) data sets to reconstruct emergent metabolic processes (Fondi and Liò, 2015). This has led to the development of new analytical platforms and methods as well as advanced computational workflows enabling more holistic understanding of metabolic expression at the individual, population, and community levels of biological organization. Nowhere is this more evident than in the rapid development of volatilome research, an extension of analytical chemistry and multi-omics integration focused on detection and quantification of volatile organic compounds (VOCs) produced by interacting cells (Mansurova et al., 2018). This current Research Topic “*Metabolomics and Transcriptomics in Biomarker Discovery: Mass Spectrometric Techniques in Volatilome Research*” presents the readers with a compilation of 10 original research articles and two contemporary reviews exploring advances in metabolomics and VOC detection. The contributions range from human medical applications related to biomarker discovery to biotechnology applications in fungi, plants and microbial cell systems.

Alodiab et al. used a metabolomics approach on dried blood samples to identify two potential biomarkers for galactosemia (GAL), a human genetic disorder that can cause life-threatening side effects related to defective glucose metabolism. Similarly, Sakanaka et al. performed metabolic profiling of saliva and plasma samples and identified a significant association between salivary allantoin and 1,5-anhydroglucitol (1,5-AG) useful in the development of a non-invasive protocol to diagnose atherosclerosis in patients with type 2 diabetes (T2D). Zhou et al. used a higher resolution method to investigate metabolite profiles associated with the treatment of cerebral ischemic stroke with *Danshen Chuanxiongqin* (DSCXQ) preparations, a traditional Chinese medicine. They identified 55 distinct metabolites involved in sphingolipid metabolism between treatment groups that were differentially impacted by DSCXQ consistent with a dampening mode of action on the neuroinflammatory response. Zhang et al. used a similar high-resolution method to profile metabolites associated with clonorchiasis (a parasitic infection caused by the Chinese liver fluke *Clonorchis sinensis*) using a rodent model system of infection. Phan and Blank investigated the fungus *Ustilago maydis* using stable-isotope labeling methods to profile metabolites produced in response to different carbon sources. They compared a range of sample preparation and processing methods to define a process for absolute quantitation in the context of bioprocess development in *Ustilago maydis*.

From the vantage of VOC detection, Furuhashi et al. devised a sampling procedure to extract VOCs from the headspace of lung cell cultures to better understand the interplay between metabolite production and cancer. They identified evidence for increased lipid peroxidation as measured in the increase of trans-2-hexenol and correlated this with changes in the levels of alcohol dehydrogenase 1C gene linking metabolite production to gene expression in cancer cell lines. Fenn et al. used a similar approach to explore the interplay between common respiratory pathogens *Staphylococcus aureus* and *Pseudomonas aeruginosa* grown in isolation or in co-culture with human lung cells. They detected compounds associated with bacterial infection as well as changes in the levels of selected VOCs based on culture conditions relevant to human *in vivo* environments. Issitt et al. measured VOCs produced by breast cancer cell lines exposed to low oxygen conditions representative of the tumor microenvironment to identify potential biomarkers in cell hypoxia. They measured increased uptake of methyl chloride, acetone and n-Hexane from hypoxic cells and concomitant production of styrene, describing volatilomic mechanisms behind this cellular condition for the first time. Expanding on the biomarker theme, Weber et al. used breath analysis combined with a machine learning approach to differentiate between the VOC profiles of children with and without asthma. They were able to discern a wide range of metabolites in the exhaled breath of asthmatic children linked to metabolic processes associated with chronic disease. Farneti et al. used a high-throughput method for rapid phenotyping of raspberry fruit maturation in relation to VOC production to assess fruit quality and to identify cultivars with optimal aroma profiles for future breeding programs.

Finally, two reviews explore methods and applications of volatilome research through a contemporary exploration of the relevant literature. Bajo-Fernández et al. consider the expanding use of exhaled breath analysis as an exciting area of clinical research with the potential to define a new era of non-invasive diagnostic testing. They highlight the need for improved standards of practice related to sample processing, VOC detection, and analysis required to achieve this vision. Szeitz et al. take a more historical approach to mass spectrometry platform innovation in the context of VOC profiling, shining a spotlight on the interrelationship between technological advances and the expansion of volatilome research into increasingly complex matrices. The resulting platform descriptions provide a useful guide intended to assist practitioners and potential end-users performing VOC analysis with advanced instrument performance. Collectively, contributions to this Research Topic capture the current state of the art in metabolite and VOC profiling, reinforcing the potential of metabolomic methods and applications in biomarker discovery and biotechnology innovation. Importantly, this is a rapidly advancing area of research that brings together the peer efforts of scientists and engineers to develop integrated platforms and workflows needed to improve mass resolution and throughput. Effective uptake of these advances will require coordinated and dedicated access to infrastructure and education across multiple training levels and disciplines.
